# The Rapunzel Syndrome: An Unusual Trichobezoar Presentation

**DOI:** 10.1155/2010/841028

**Published:** 2010-03-24

**Authors:** Luiz Roberto Lopes, Priscilla Sene Portel Oliveira, Eduardo Marcucci Pracucho, Marcelo Amade Camargo, João de Souza Coelho Neto, Nelson Adami Andreollo

**Affiliations:** Department of Surgery, School of Medicine, University of Campinas (Unicamp), Rua Tessália Vieira de Camargo, 126 - Cidade Universitária Zeferino Vaz, Campínas - SP - CEP: 13083-887, Brazil

## Abstract

The Rapunzel syndrome is an unusual form of trichobezoar found in patients with a history of psychiatric disorders, trichotillomania (habit of hair pulling) and trichophagia (morbid habit of chewing the hair), consequently developing gastric bezoars. The principal symptoms are vomiting and epigastric pain. In this case report, we describe this syndrome in a young girl.

## 1. Introduction

This syndrome is named after the girl with the long tresses in the fairy tale written by the Grimm Brothers in 1812. The Rapunzel syndrome was first reported in the literature by Vaughan et al. in 1968 [1] and was so named because the length of the hair and the uncommonness of the situation are characteristics both of the fairy tale and of the clinical cases described in the report published by these investigators.

For centuries bezoars have been known to occur in the form of undigested masses found in the stomach of animals and humans. Nevertheless, this finding has become more common in humans as a result of more frequent manipulation of the gastrointestinal tract [2]. From the increase in the incidence of this finding and the current ease with which knowledge is disseminated today, it is clear that the Rapunzel syndrome, two cases of which were reported by Vaughan et al., remains rare, a finding of phytobezoars being more common [2]. This fact was recently confirmed in a literature review performed in 2007 that identified 27 cases of Rapunzel syndrome described between 1968 and 2006 [3]. A few other reports on this syndrome were published in the medical literature after this date [4–6].

There are several different forms of presentation of this syndrome; however, in general, it involves the presence of a gastric trichobezoar with a long tail extending beyond the duodenum, as found in the case reported here. The factor responsible for this syndrome is the compulsion of patients to pull out their own hair and swallow it, processes referred to as trichotillomania and trichophagia, disorders that affect young girls with or without known psychiatric disorders [3, 4].

## 2. Case Report

Female, 22 years old, was referred to a gastroenterology surgical team for evaluation following a history of vomiting over the past three months together with continuous, intense epigastric pain. In addition, the patient had an upper digestive tract endoscopy that revealed a tumor consisting of strands of black hair extending towards the antrum and blocking visualization of the pylorus.

The patient was slightly pale, this being the only abnormality found at physical examination. She had a history of slightly retarded neuropsychomotor development. The patient's mother admitted that she had a habit of pulling out her hair and secretly swallowing it. She was admitted to hospital for surgical treatment and psychiatric evaluation.

**Figure 1:**
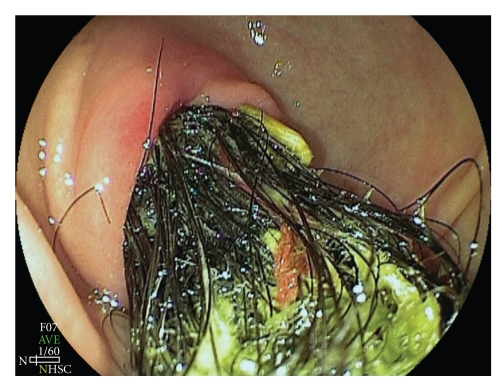
Endoscopic image of a trichobezoar extending into the pylorus.

**Figure 2:**
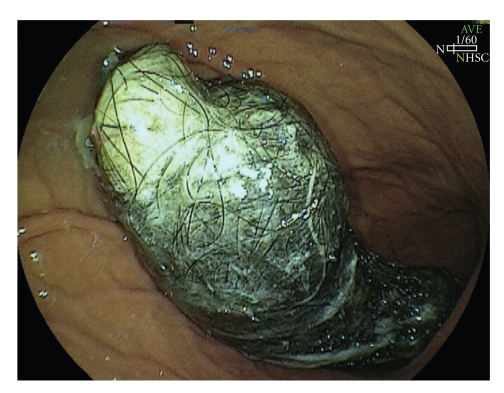
Endoscopic image of an intragastric trichobezoar.

**Figure 3:**
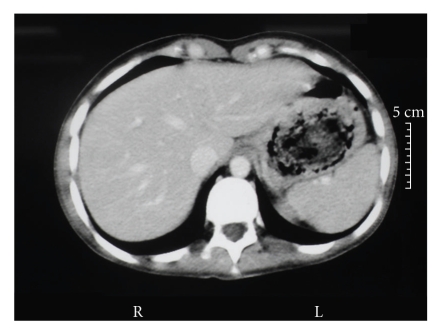
Tomography image of a trichobezoar in the stomach.

**Figure 4:**
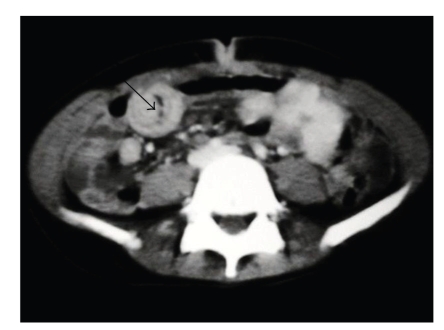
Tomography image of bowel invagination (arrow).

A second endoscopy of the upper digestive tract was performed as part of the preoperative work-up, findings revealing a blackened, voluminous mass composed of strands of hair, fibrin and food residue in the stomach, located in the antrum and low body, extending towards the pylorus and blocking passage of the endoscope (Figures 1 and 2). A computed tomography scan of the abdomen was also performed and, in addition to identifying a hypodense intragastric mass, results showed hypodense circular areas in the small bowel and the presence of bowel intussusception (Figures 3 and 4), findings compatible with the Rapunzel syndrome [7].

**Figure 5:**
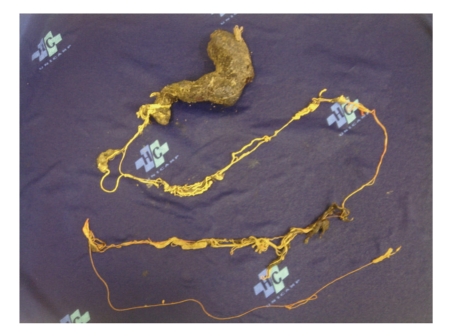
Trichobezoar and strands of 1.6 meters in length removed at surgery.

Surgical treatment was initiated by videolaparoscopy, which was then converted to laparotomy due to the finding of a large intragastric mass that could not be removed by the technique initially selected. Gastrotomy was performed and the trichobezoar was removed together with strands in the shape of the stomach and the first portion of the duodenum, extending through the small intestine to a total length of 1.6 meters (Figure 5). 

In addition, bowel intussusception was found one meter from the angle of Treitz, which was unravelled manually, and a trichobezoar of two centimeters in diameter was found in the proximal ileum extending as far as the ascending colon.

The patient progressed with no complications and was discharged from hospital five days after surgery. She is currently being followed up as an outpatient by the gastroenterology, surgical and psychiatric teams.

## 3. Discussion

It has already been confirmed in the literature that the Rapunzel syndrome occurs predominantly in young women with psychiatric disorders [3, 7], and consists of the presence of a rare type of trichobezoar [5]. The patient in this report had been diagnosed with mild mental retardation. Trichotillomania and trichophagia were only reported following the physician's insistence during anamnesis after noticing that the patient had some bald patches on her scalp resulting from the habit of pulling out her hair and secretly swallowing it [8].

The diagnosis was made by upper gastrointestinal tract endoscopy, indicated because of the patient's history of vomiting and persistent weight loss, and confirmed by computed tomography, which revealed the extent of the trichobezoar in the stomach and small bowel and also confirmed the suspicion of bowel invagination, a relatively common finding in the presence of a bezoar, occurring in around 7% of all reported cases [3]. This was confirmed during inspection and was able to be unravelled easily. Both abdominal ultrasonography and computed tomography are capable of detecting a bezoar [7]. Obstruction of the upper digestive tract is the most common clinical manifestation of this disorder [4].

Options for the treatment of trichobezoars include the use of chemical substances in the stomach to try to dissolve the material, and mechanical fragmentation. However, in the case of larger trichobezoars, these methods are less likely to be successful [5, 6]. Smaller trichobezoars may be removed by endoscopy.

When the loop is damaged showing necrosis or perforation, which has been described in some cases, bowel resections may have to be performed [9]. Other manifestations include upper digestive tract bleeding, anemia and bowel intussusception. Death is rare [5, 6].

Regarding optimal surgical management of these cases, new reports on the use of videolaparoscopy are encouraging. However, sample sizes have so far been small and, for this reason, the role of this technique in the treatment of the Rapunzel syndrome remains to be defined [10]. Nevertheless, use of videolaparoscopy should not be discarded until its possible role has been firmly established, since this technique has been shown to be advantageous in various other surgical procedures in the gastrointestinal tract. In the present case, laparoscopy was attempted; however, due to the large size of the trichobezoar, it was found to be impossible to remove it by this route. The stomach cavity had to be opened and gastrotomy was performed on the anterior wall of the stomach, as has been described in the majority of papers [5, 11, 12].

Psychiatric followup is important although no conclusion has been reached with respect to whether the use of medication makes any difference in the progression of this condition [8]. This followup care should be extended to family members, who should be vigilant with patients, since recurrences of the problem have been described [8]. The need for adequate follow-up should be emphasized to avoid recurrences, although these are rare since the trauma of surgery may prevent the patient from provoking another episode [6].

The Rapunzel syndrome is rare but should be taken into consideration in investigating cases of female patients with a history of vomiting, weight loss and anemia. Diagnostic workup should include upper digestive tract endoscopy, ultrasonography and abdominal tomography. Treatment is surgical in most cases. Complications resulting from perforation or bowel necrosis may occur but death is rare.
